# Characterization of lipid droplet metabolism patterns identified prognosis and tumor microenvironment infiltration in gastric cancer

**DOI:** 10.3389/fonc.2022.1038932

**Published:** 2023-01-11

**Authors:** Mengxiao Liu, Xidong Fang, Haoying Wang, Rui Ji, Qinghong Guo, Zhaofeng Chen, Qian Ren, Yuping Wang, Yongning Zhou

**Affiliations:** ^1^ The First Clinical Medical College, Lanzhou University, Lanzhou, China; ^2^ Department of Gastroenterology, the First Hospital of Lanzhou University, Lanzhou, China; ^3^ Key Laboratory for Gastrointestinal Diseases of Gansu Province, The First Hospital of Lanzhou University, Lanzhou, China; ^4^ Department of Gastroenterology, Tangdu Hospital, Fourth Military Medical University, Xinan, China

**Keywords:** lipid droplet metabolism, gastric cancer, tumor immunity, subtypes, prognostic model

## Abstract

**Background:**

Gastric cancer is one of the common malignant tumors of the digestive system worldwide, posing a serious threat to human health. A growing number of studies have demonstrated the important role that lipid droplets play in promoting cancer progression. However, few studies have systematically evaluated the role of lipid droplet metabolism-related genes (LDMRGs) in patients with gastric cancer.

**Methods:**

We identified two distinct molecular subtypes in the TCGA-STAD cohort based on LDMRGs expression. We then constructed risk prediction scoring models in the TCGA-STAD cohort by lasso regression analysis and validated the model with the GSE15459 and GSE66229 cohorts. Moreover, we constructed a nomogram prediction model by cox regression analysis and evaluated the predictive efficacy of the model by various methods in STAD. Finally, we identified the key gene in LDMRGs, ABCA1, and performed a systematic multi-omics analysis in gastric cancer.

**Results:**

Two molecular subtypes were identified based on LDMRGs expression with different survival prognosis and immune infiltration levels. lasso regression models were effective in predicting overall survival (OS) of gastric cancer patients at 1, 3 and 5 years and were validated in the GEO database with consistent results. The nomogram prediction model incorporated additional clinical factors and prognostic molecules to improve the prognostic predictive value of the current TNM staging system. ABCA1 was identified as a key gene in LDMRGs and multi-omics analysis showed a strong correlation between ABCA1 and the prognosis and immune status of patients with gastric cancer.

**Conclusion:**

This study reveals the characteristics and possible underlying mechanisms of LDMRGs in gastric cancer, contributing to the identification of new prognostic biomarkers and providing a basis for future research.

## Introduction

Gastric cancer is one of the most common malignant tumors of the digestive tract worldwide, with the fifth highest incidence and the fourth highest mortality rate (1). Early gastric cancer is mainly treated with surgery, adjuvant chemotherapy and radiotherapy, while chemotherapy remains the main treatment for advanced gastric cancer. Although, with the development of molecular biology of tumors, molecular targeted therapy and immunotherapy have achieved some success in the treatment of advanced gastric cancer (2), only a small percentage of patients can benefit from them and most patients with advanced gastric cancer still have a poor prognosis. Therefore, there is an urgent need to find new biomarkers to construct clinical prediction models for risk stratification and outcome prediction in patients with gastric cancer.

Lipid droplets are an evolutionarily highly conserved organelle consisting of a single phospholipid membrane wrapped around a core of neutral lipids involved in the storage and utilization of lipids (3). Recent studies have shown that in addition to adipocytes, lipid droplets have also been found in various cells such as hepatocytes, smooth muscle cells and glial cells. These findings clarify that lipid droplets do not only serve as storage sites for neutral lipids, but also have various functions such as inhibition of metabolism and regulation of gene expression (4, 5). In addition to their lipid and cholesterol storage functions, lipid droplets have recently been found to be associated with inflammatory responses, obesity, atherosclerosis and cancer (6–8).

A growing number of studies have demonstrated that the gradual accumulation of lipid droplets is a distinctive feature of many types of cancer (9–11). These lipid droplets store excess lipids to avoid lipotoxicity and can provide sufficient raw material for biofilms for the proliferation of cancer cells. In addition, lipid droplets provide a sufficient source of energy for tumor invasion and are associated with chemotherapy resistance (12, 13). In addition, lipid droplets can be used as a controlled and biocompatible vehicle for the delivery of anticancer drugs (14). Therefore, targeting altered lipid droplet metabolic pathways is a promising anti-cancer strategy (15).

The development of gastric cancer is closely related to lipid droplet metabolism. A study has shown that inhibition of DGAT2 expression enhances the sensitivity of gastric cancer to anoikis *in vitro* and inhibits peritoneal metastasis *in vivo* by disrupting lipid droplet formation in a lipid-rich environment (16). Furthermore, studies have confirmed the accumulation of lipid droplets that do exist in gastric epithelial tumors, further demonstrating the close relationship between lipid droplet metabolism and gastric cancer (17). Nevertheless, the expression patterns and functions of LDMRGs in STAD remain to be systematically analyzed.

In this study, we systematically analyzed multi-omics data from LDMRGs and identified 2 subtypes of STAD with different survival prognostic and immunological features. In addition, lasso regression models and nomogram prediction models were constructed based on the expression profiles of LDMRGs, which have reliable predictive efficacy for OS of patients with STAD by risk score. Moreover, we took the intersection of hub genes and the results of multivariate cox regression analysis to identify the key gene of LDMRGs, ABCA1. Finally, we performed a systematic multi-omics analysis of ABCA1 in STAD, and the results demonstrated that ABCA1 can predict outcomes in patients with STAD and has the potential to be a new therapeutic target for STAD.

## Materials and methods

### Data collection and process

RNA-sequence data (375 tumors and 32 normal, TPM value), genetic mutation and corresponding clinical information of stomach adenocarcinoma (STAD) were downloaded from The Cancer Genomics Atlas (TCGA) dataset (https://portal.gdc.com) (18). The GSE15459 cohort, GSE66229 cohort and GSE26253 cohort were downloaded from Gene Expression Omnibus (GEO) database (http://www.ncbi.nih.gov/geo) (19).

### Gene expression analysis

Lipid droplet metabolism-related genes were obtained from the GeneCards (https://www.genecards.org/) database (20) by searching for the keyword “Lipid droplet metabolism” and filtering for “relevance score>40”. The detailed information on LDMRGs can be found in **Supplementary Table 1**. We analyzed the differential expression and correlation of 21 LDMRGs in TCGA-STAD. In addition, we analyzed the differential expression of ABCA1, a hub gene in LDMRGs, in STAD by combining the TCGA-STAD cohort and the Genotype-Tissue Expression (GTEx) database (21). These analyses were performed statistically using the R software (version 3.6.3) and the ggplot2 package (version 3.3.3) was mainly used for visualization. Moreover, we analyzed the differential expression of ABCA1 in gastric cancer of gene chip data from GEO using TNMplot platform (https://tnmplot.com/analysis/) (22).

### Unsupervised clustering for 21 lipid droplet metabolism-related genes

Consistency analysis was performed using ConsensusClusterPlus R package (v1.54.0) with a maximum number of clusters of 6 and 100 replicates to extract 80% of the total sample, clusterAlg = “hc”, innerLinkage = “ ward.D2” (23). The clustering heatmaps were all analysed by the R software package complex heatmap (v2.2.0), and gene expression heatmaps were retained for genes with variance above 0.1. PCA plots were plotted using the ggord package and OPLS-DA analysis was performed using the Metware Cloud, a free online platform for data analysis (https://cloud.metware.cn).

### Construction of lasso regression models and nomogram models

The least absolute shrinkage and selection operator (LASSO) regression algorithm was used for feature selection, 10-fold cross-validation was used, and the glmnet package (version 4.1-2) and the survival package (version 3.2-10) were used for the analysis. Log-rank test was used to compare differences in survival between two groups. The univariate Cox regression analysis was conducted to identify proper terms for the construction of the nomogram. The multivariate Cox regression analysis was performed to further identify independent prognostic factors for STAD. The rms package (version 6.2-0) and survival package (version 3.2-10) were used to create nomogram to predict the total recurrence rate in 1, 3, and 5 years. The timeROC(version 0.4) analysis was used to compare the predictive accuracy of risk score. The survival package (version 3.2-10) and stdca.R files (24) were used to create DCA curves to assess the clinical utility value of the model.

### The protein-protein interactions analysis

We analyzed the protein-protein interactions between LDMRGs through the STRING (https://cn.string-db.org/) database (25). Moreover, we used cytoscape’s cytoHubba plugin to obtain the top five hub genes in LDMRGs by the MCC scoring method. Finally, we obtained two important functional modules through the MCODE plug-in in the cytoscape software.

### Gene function enrichment analysis

GO and KEGG analyses were performed using R software with a cutoff p value <0.05 and an adjusted p value <0.1. The clusterProfiler (26) package (version 3.14.3) was used for enrichment analysis and the org.Hs.eg.db package (version 3.10.0) was used for ID conversion. Gene Set Enrichment Analysis (GSEA) was performed using CAMOPI (https://www.camoip.net/) database (27).

### Analysis of differentially expressed genes

The differential expression of mRNA was identified using the limma package for R software (version 3.14.3). “ adjusted p value < 0.05 and log2 (fold change) > 1.5 or log2 (fold change) < -1.5” was defined as the threshold for the differential expression of mRNAs.

### Analysis of genetic alterations

LDMRGs alterations were analyzed using the cBioPortal (28) database (http://www.cbioportal.org/). ABCA1 mutation analysis was performed primarily using the maftools package in R software to download and visualize somatic mutations in patients with STAD.

### Correlation between LDMRGs mRNA expression levels and clinical characteristics, prognosis, copy number variation, and methylation in STAD

The relationship between ABCA1 mRNA expression level and clinical characteristics in TCGA-STAD cohort was analyzed using a dichotomous logistic model constructed with R software. The diagnostic value of ABCA1 mRNA expression level for gastric cancer was analyzed by the receiver operating characteristic (ROC) curves using the R packages mainly the pROC package (version 1.17.0.1) and the ggplot2 package. The relationship between LDMRGs mRNA expression levels and prognosis was mainly analysed statistically and visualised using the survminer package (version 0.4.9) and the survival package (version 3.2-10) for survival data. Correlation of LDMRGs mRNA expression levels with CNV and methylation was analyzed by the Gene Set Cancer Analysis (GSCA) (29) database (http://bioinfo.life.hust.edu.cn/GSCA/#/).

### Analysis of immune infiltration and immune checkpoint blockade treatment

The correlation between LDMRGs mRNA expression levels and immune infiltration was mainly analyzed using the GSCA database. The comparison of immune cell infiltration levels between different subgroups was analysed using the immunedeconv package, which integrates six state-of-the-art algorithms including TIMER, xCell, MCP-counter, CIBERSORT, EPIC and quanTIseq. Then, the results were visualised using the ggplot2 package. Moreover, we analyzed the differential expression of 8 immune checkpoint-related genes in different subgroups using the ggplot2 package. Finally, we used the Tumor Immune Dysfunction and Exclusion (TIDE) algorithm (30) to predict the responsiveness of different subtypes to immune checkpoint inhibitors based on gene expression profiling data. The results of the analysis were visualized using the ggplot2 package and the ggpubr package (0.4.0). In addition, we analyzed the differences in the level of infiltration of different immune cells between the high and low ABCA1 expression groups using the ssGSEA algorithm built into the GSVA package (version 1.34.0), and also calculated the differences between the stromal score, immune score and estimate score between the different groups using the estimate package (version 1.0.13) (31). The relationship between ABCA1 expression levels and different molecular subtypes and immunological subtypes was analyzed by the TISIDB (32) database (http://cis.hku.hk/TISIDB/). The relationship between ABCA1 expression levels and TMB, MSI and Neoantigen Loads was analyzed using the CAMOIP database.

### Comparison of differences in m6A-related gene expression levels and differences in stemness scores in different subgroups

The m6A-related genes were derived from past study (33) and the heat map of m6A-related gene expression between different subgroups in the TCGA-STAD cohort was visualized using the complex heatmap package. Furthermore, the OCLR algorithm constructed by Malta et al. was used to calculate mRNAsi and to assess the degree of stemness of samples in different subgroups (34, 35).

### Correlation analysis of ABCA1 expression levels and drug sensitivity

Correlation analysis of mRNA expression levels of ABCA1 and CTRP drug sensitivity was performed using the drug module of the GSCA database.

### Statistical analysis

T test was used when the two groups met normal distribution and homogeneity of variance; Wilcoxon rank sum test was used when the two groups did not meet normal distribution. Kaplan-meier method was used for survival curve analysis of prognosis. and median gene expression level was used for grouping. Logrank test or Cox regression was used for differences between groups. The ROC curve was used to evaluate the predictive effectiveness of the model. Two-tailed p < 0.05 was considered statistically significant (ns: p > 0.05, *: p ≤ 0.05, **: p ≤ 0.01, ***: p ≤ 0.001, ****: p ≤ 0.0001).

## Results

### LDMRGs expression and functional enrichment analysis in STAD

**Figure 1:**
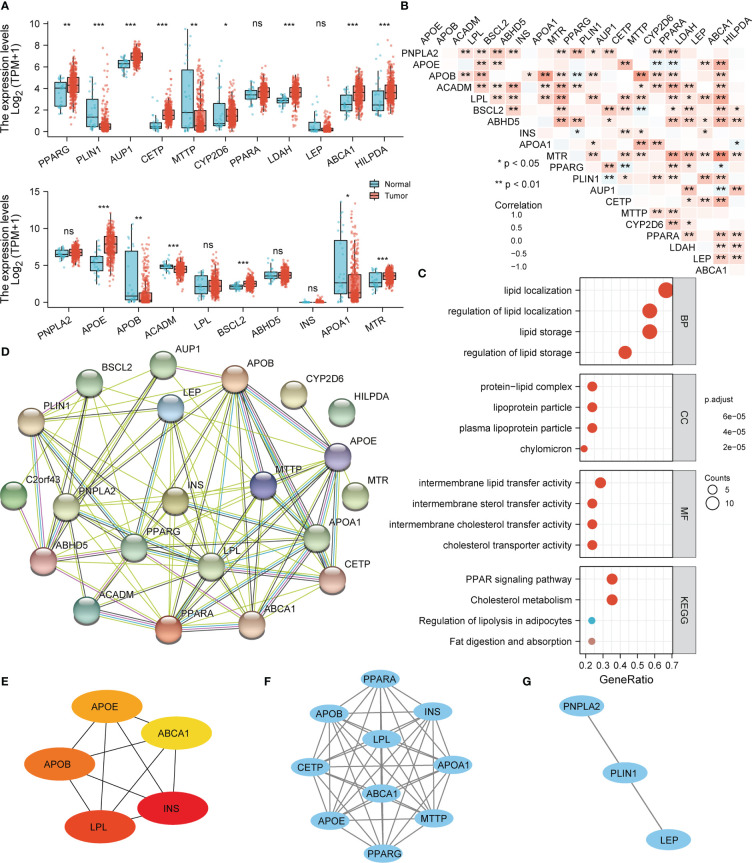
The differential expression and functional enrichment analysis of LDMRGs in STAD. **(A)** Differential expression analysis of LDMRGs in STAD. **(B)** Correlation analysis of the expression of LDMRGs in STAD. **(C)** GO and KEGG pathway enrichment analysis of LDMRGs. **(D)** Construction of PPI interaction network for LDMRGs using the STRING database. **(E)** Identification of the top 5 hub genes in the PPI Interaction network of LDMRGs by cytoscape software. **(F, G)** Identification of key network modules in PPI interaction network for LDMRGs *via* cytoscape software. (ns: p > 0.05,*: p ≤0.05, **: p ≤ 0.01, ***: p ≤ 0.001).

We explored the differential expression of LDMRGs in the TCGA-STAD cohort and as shown in **Figure 1A**, 10 LDMRGs were highly expressed in STAD, including PPARG, AUP1, CETP, CYP2D6, LDAH, ABCA1, HILPDA, APOE BSCL2, and MTR. 5 LDMRGs were lowly expressed in STAD, including PLIN1, MTTP, APOB, ACADM, and APOA1. We then performed a correlation analysis based on the expression levels of LDMRGs and the results showed a general positive correlation between the expression levels of LDMRGs in STAD (**Figure 1B**). Moreover, we performed GO and KEGG pathway enrichment analysis on LDMRGs. As shown in **Figure 1C**, these genes were mainly involved in lipid storage and transport, fat digestion and absorption, and cholesterol metabolism. To further explore the interactions between these genes, we constructed a PPI network using the STRING database (**Figure 1D**) and then identified the top 5 hub genes using cytoscape, including: INS, LPL,APOB,APOE, and ABCA1 (**Figure 1E**). Then 2 key sub-networks were identified through the MCODE plugin (**Figures 1F, G**).

### Analysis of genetic alterations and correlation between mRNA expression of LDMRGs and CNV, methylation and immune infiltration in STAD

**Figure 2:**
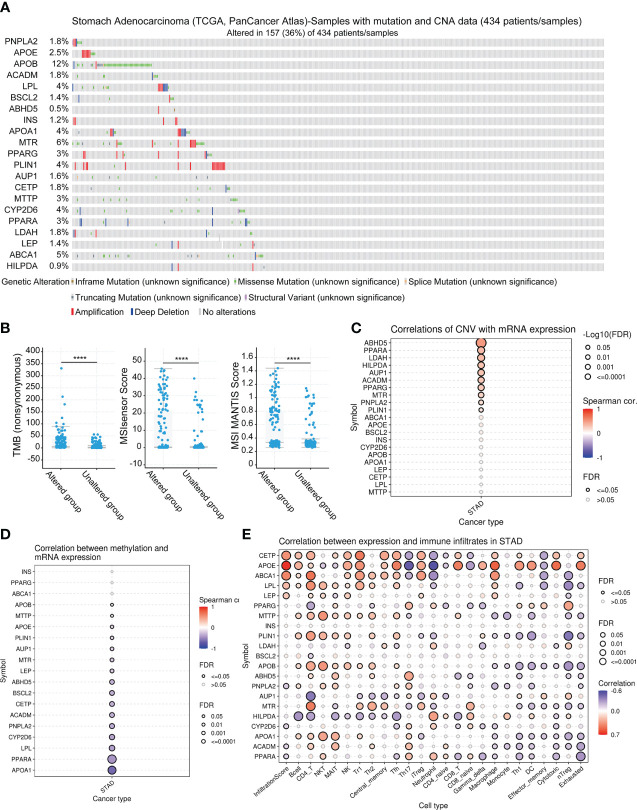
Multi-omics analysis of LDMRGs in STAD. **(A)** Landscape analysis of genetic alterations of LDMRGs in STAD using the cBioPortal database. **(B)** Differential analysis of TMB, MSI in different genetic alteration groups in STAD using the cBioPortal database. **(C–E)** Correlation analysis of mRNA expression levels of LDMRGs and CNV, methylation, and immune infiltration levels in STAD. (****: p ≤ 0.0001).

We analyzed genetic alterations in the TCGA-STAD, PanCancer Atlas cohort using the cBioPortal database. As shown in **Figure 2A**, the total frequency of genetic alteration was 36% (157/434), with the top 3 genes most frequently altered being APOB (12%), MTR (6%), and ABCA1 (5%). The types of genetic alterations were mainly missense mutation, amplification, and truncating mutation. Furthermore, we noted that TMB and MSI were significantly higher in the genetically altered group than in the unaltered group, implying that patients in the genetically altered group may be more effective in immunotherapy for STAD (**Figure 2B**). In addition, we explored that LDMRGs mRNA expression was generally positively correlated with CNV, negatively correlated with methylation levels, and strongly correlated with multiple immune cell infiltrations (**Figures 2C–E**). These results indicated that LDMRGs may be involved in the progression of STAD through genetic alterations and immune regulation.

### Identification of two clusters by consensus clustering of LDMRGs in STAD

**Figure 3:**
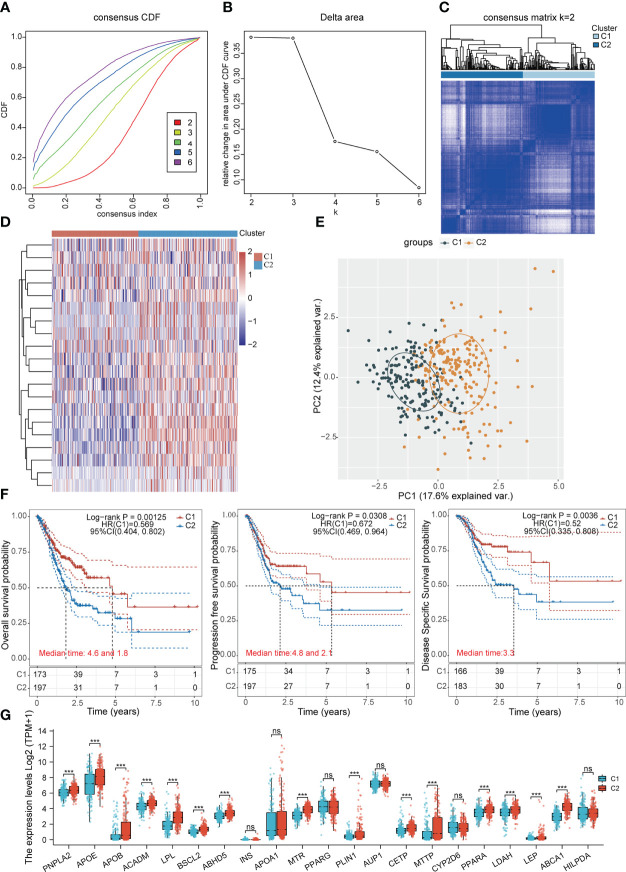
Identification of subtypes associated with LDMRGs in STAD. **(A–C)** The optimal number of clusters (K=2) was determined for classification based on the cumulative distribution function (CDF) curve. **(D)** Heat map of the expression of LDMRGs in different subgroups, red represents high expression and blue represents low expression. **(E)** Principal component analysis (PCA) of 375 patients with STAD, with each point representing one sample. **(F)** Survival analysis between different subgroups in the TCGA-STAD cohort, including OS, PFS, and DSS. **(G)** Differential expression analysis of LDMRGs between different subgroups in the TCGA-STAD cohort. (ns: p > 0.05, *: p ≤0.05, **: p ≤ 0.01, ***: p ≤ 0.001).

Based on the expression levels of LDMRGs, the TCGA-STAD samples can be classified into 2 molecular subtypes, cluster1 (C1) and cluster2 (C2), using an unsupervised clustering method (**Figures 3A–E**). In addition, we performed a supervised OPLS-DA analysis, as shown in **Supplementary Figure 2**, which also distinguished C1 and C2 subtypes better. Then, we analyzed the survival prognosis between the 2 different molecular subtypes by kaplan-Meier (KM) curve, as shown in **Figure 3F**, subtype C2 had worse OS prognosis, progression-free survival (PFS) prognosis and disease-specific survival (DSS) prognosis compared with subtype C1. Interestingly, we found that most LDMRGs were expressed at higher levels in C2 subtype compared with C1 subtype, including PNPLA2, APOE, APOB, ACADM, LPL, BSCL2, ABHD5, MTR, PLIN1, CETP, MTTP, PPARA, LDAH, LEP, and ABCA1 (**Figure 3G**). This could mean that high expression of these LDMRGs may be associated with a poorer prognosis for patients in STAD.

**Figure 4:**
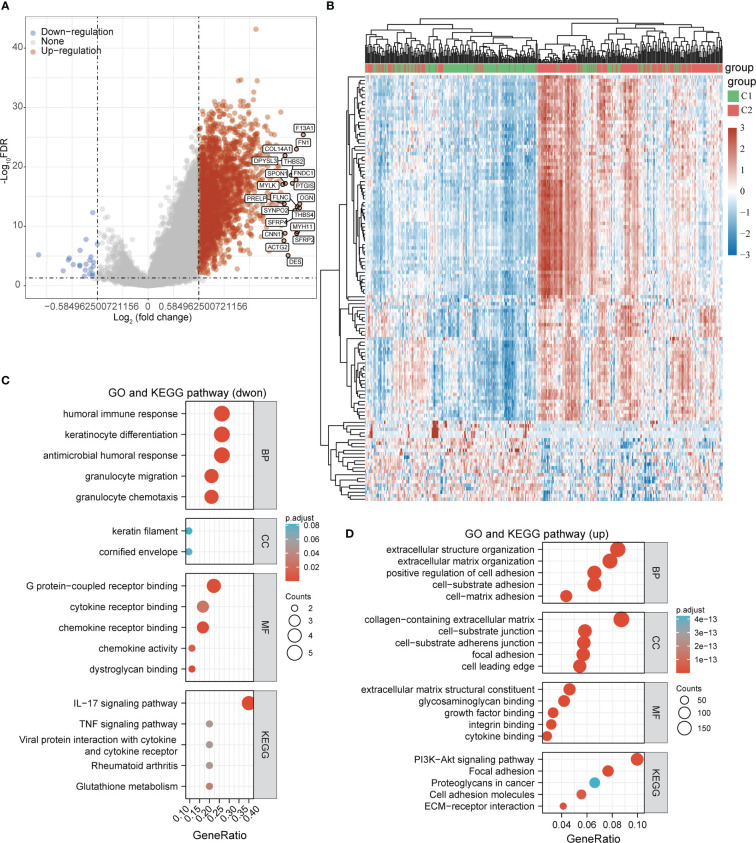
Functional enrichment analysis of DEGs between the two subgroups. **(A, B)** Volcano and heat maps of differentially expressed genes between the two subgroups in the TCGA-STAD cohort. **(C)** GO and KEGG pathway enrichment analysis of differentially expressed genes that were dwon-regulated. **(D)** GO and KEGG pathway enrichment analysis of differentially expressed genes that were up-regulated.

To further analyze the molecular functional differences between the 2 subtypes, we performed differential gene analysis on the 2 subtypes and obtained a total of 2257 differentially expressed genes that were up-regulated and 22 differentially expressed genes that were down-regulated in STAD (**Figures 4A, B**). The GO and KEGG pathway enrichment analysis revealed that these differentially expressed genes were mainly involved in immune regulation, inflammatory response, and glutathione metabolism. The up-regulated differential genes were mainly involved in cell adhesion, extracellular matrix composition, and PI3K-Akt signalling pathway (**Figures 4C, D**). We speculated that differences in immunomodulation and cell adhesion led to different survival prognosis between these 2 subgroups in STAD.

### Correlation analysis between subtypes and immune infiltration, ICB treatment, m6A methylation modification, and tumour stemness

**Figure 5:**
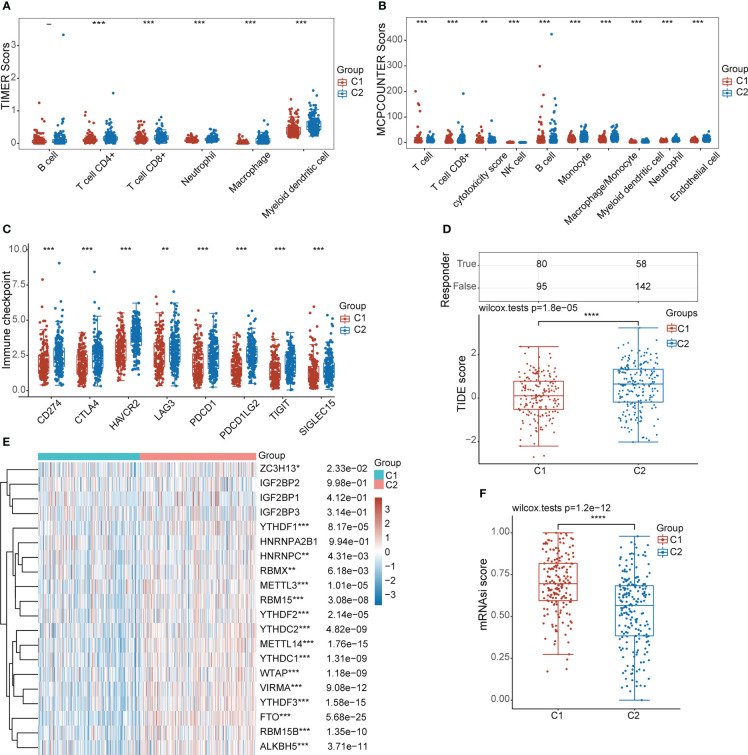
Characteristic analysis of different subgroups in the TCGA-STAD cohort. **(A, B)** Analysis of differences in the level of immune infiltration between the two subgroups. **(C)** Differential expression analysis of immune checkpoint-related genes between the two subgroups. **(D)** Analysis of the differences in TIDE scores between the two subgroups. **(E)** Heat map of differential expression of m6A methylation-related genes between the two subgroups. **(F)** Analysis of differences in tumor stemness scores between the two subgroups. (*: p ≤0.05, **: p ≤ 0.01, ***: p ≤ 0.001, ****: p ≤ 0.0001).

To further evaluate the immune status of the different subgroups, we compared the differences in the infiltration levels of immune cells in the 2 subgroups by the TIMER and MCP-counter algorithms. As shown in **Figures 5A, B**, the level of infiltration of multiple immune cells was significantly higher in the C2 subgroup than in the C1 subgroup. Furthermore, we assessed the expression levels of immune checkpoint-related genes in different subgroups. Interestingly, we also found that the expression levels of immune checkpoint-associated genes were significantly higher in the C2 subgroup than in the C1 subgroup (**Figure 5C**). TIDE algorithm was used to predict the response of the two subgroups to ICB treatment, and the results showed that C2 subgroup had a lower TIDE score, indicating that C2 subgroup had a better effect on ICB treatment (**Figure 5D**). Past studies have shown that m6A methylation modifications (36, 37) and tumour stemness (38, 39) are involved in the progression of a variety of tumours. Our study showed that the expression levels of most key genes associated with m6A methylation modifications were higher in the C2 subgroup than in the C1 subgroup. However, the stemness index of the C2 subgroup was lower than that of the C1 subgroup (**Figures 5E, F**). The relationship between lipid droplet metabolism and m6A methylation modifications and tumour stemness in STAD needs to be further investigated.

### Construction and validation of the lasso regression model

**Figure 6:**
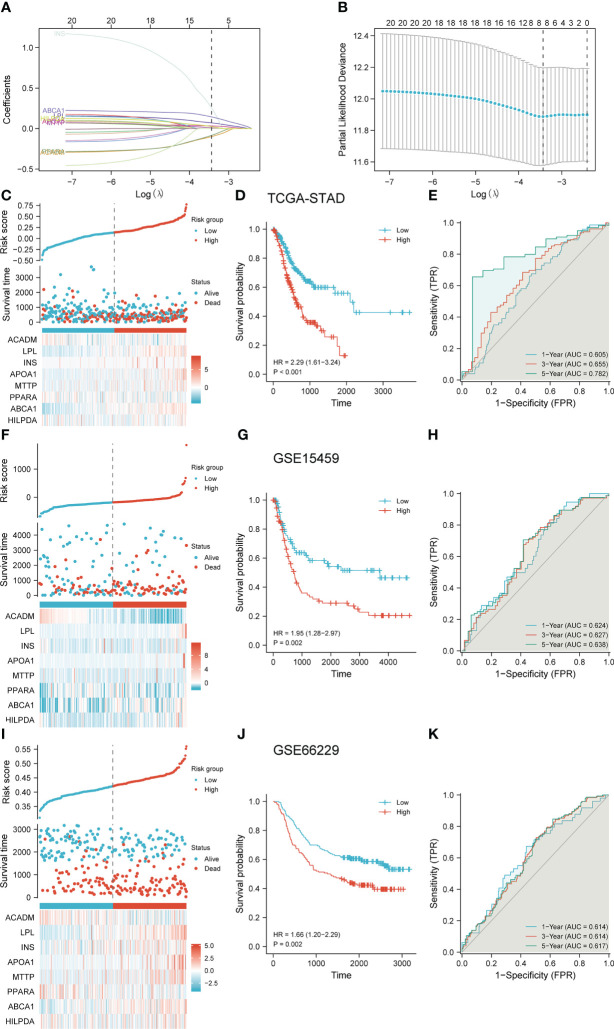
Construction and validation of the Lasso regression model. **(A)** Trajectory plots of variables for Lasso regression analysis. **(B)** Screening of coefficients for Lasso regression analysis variables. **(C–E)** Construction and evaluation of a risk prediction scoring system in the TCGA-STAD cohort. **(F–H)** Validation and evaluation of risk prediction scoring systems in the GSE15459 cohort. **(I–K)** Validation and evaluation of risk prediction scoring systems in the GSE66229 cohort.

To further investigate the relationship between LDMRGs and prognosis, we screened 8 key genes by lasso regression analysis and constructed a prognostic model in STAD (**Figures 6A, B**). Risk score = (-0.099 * ACADM) + (0.070 * LPL) + (0.255 * INS) + (0.032 * APOA1) + (0.009 * MTTP) + (-0.084 * PPARA) + (0.144 * ABCA1) + (0.025 * HILPDA). Then, we divided the patients in the TCGA-STAD cohort into high-risk and low-risk groups based on risk scores from the prognostic model and showed that patients in the high-risk group had a worse overall survival prognosis (**Figures 6C, D**). The ROC curves showed that the model had certain predictive efficacy for 1, 3, and 5-year survival of patients in the TCGA-STAD cohort (**Figure 6E**). In addition, to demonstrate the reliability and applicability of the model, 2 gastric cancer cohorts (GSE15459, GSE66229) from the GEO data were used to validate the prediction model, which also proved to be able to differentiate well between the high-risk and low-risk groups of patients with gastric cancer. The ROC curves showed that the model was effective in predicting survival at 1,3, and 5 years (**Figures 6F–K**).

### Construction and evaluation of nomogram model

To further explore the clinical factors related to the OS prognosis of patients with STAD, we included the 7 key genes screened by the above lasso regression analysis (due to the expression level of INS in more than half of the samples in TCGA-STAD cohort was 0, it was not possible to separate the high and low expression groups for the next cox regression analysis) and a variety of clinical characteristics in the univariate and multifactorial cox regression analysis. As shown in **Table 1**, age, T-stage, N-stage, M-stage, pathological stage, LPL, APOA1, and ABCA1 were associated with the OS prognosis of patients in the TCGA-STAD cohort. In addition, age, M-stage, and ABCA1 were independent risk factors affecting the OS prognosis of patients with STAD.

**Table 1:** Univariate and multivariate cox regression analyses were based on multiple clinical characteristics and LDMRGs in the TCGA-STAD cohort.

Characteristics	Total(N)	Univariate analysis		Multivariate analysis	
		Hazard ratio (95% CI)	P value	Hazard ratio (95% CI)	P value
Age	367				
<=65	163	Reference			
>65	204	1.620 (1.154-2.276)	**0.005**	1.979 (1.353-2.894)	**<0.001**
Gender	370				
Female	133	Reference			
Male	237	1.267 (0.891-1.804)	0.188		
T stage	362				
T1&T2	96	Reference			
T3&T4	266	1.719 (1.131-2.612)	**0.011**	1.247 (0.731-2.128)	0.419
N stage	352				
N0&N1	204	Reference			
N2&N3	148	1.650 (1.182-2.302)	**0.003**	1.175 (0.719-1.918)	0.520
M stage	352				
M0	327	Reference			
M1	25	2.254 (1.295-3.924)	**0.004**	2.133 (1.068-4.260)	**0.032**
Pathologic stage	347				
Stage I&Stage II	160	Reference			
Stage III&Stage IV	187	1.947 (1.358-2.793)	**<0.001**	1.370 (0.765-2.455)	0.290
Histologic grade	361				
G1&G2	144	Reference			
G3	217	1.353 (0.957-1.914)	0.087	1.267 (0.849-1.891)	0.246
ACADM	370	0.858 (0.673-1.094)	0.217		
LPL	370	1.179 (1.038-1.339)	**0.011**	1.032 (0.887-1.200)	0.683
APOA1	370	1.067 (1.009-1.129)	**0.023**	1.034 (0.965-1.108)	0.337
MTTP	370	1.094 (0.989-1.210)	0.080	1.061 (0.939-1.198)	0.342
PPARA	370	0.865 (0.677-1.105)	0.246		
ABCA1	370	1.303 (1.086-1.563)	**0.004**	1.283 (1.020-1.612)	**0.033**
HILPDA	370	1.108 (0.953-1.288)	0.184		

Bold values are used to highlight p-values less than 0.05.

**Figure 7:**
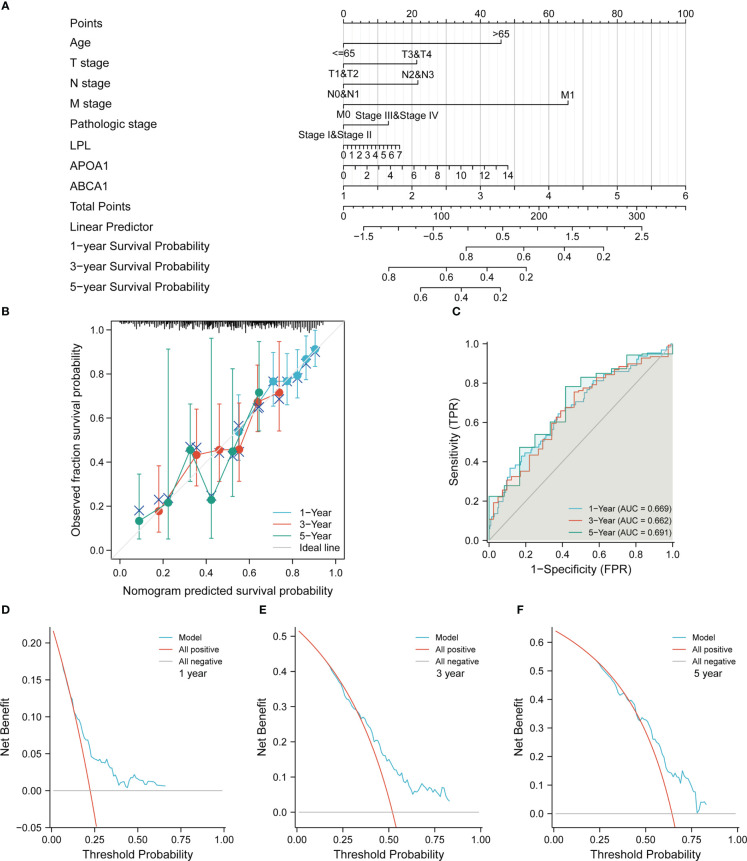
Construction and evaluation of nomogram prediction models in the TCGA-STAD cohort. **(A)** Construction of a nomogram prediction model. **(B)** Evaluation of calibration curve on the predictive value of the nomogram model. **(C)** Evaluation of ROC curve on the predictive value of the nomogram model. **(D–F)** Evaluation of DCA curve on the clinical utility value of the nomogram model.

To better assess the OS prognosis of clinical patients with STAD, we constructed nomogram prognostic model based on the results of cox regression analysis (**Figure 7A**). The Calibration curve and the ROC curve showed that the nomogram model had certain prediction efficiency for 1 -, 3 - and 5-year survival rate of patients with STAD (**Figures 7B, C**). Finally, the DCA curves demonstrated that the model also had good clinical utility in STAD (**Figures 7D–F**).

### Correlation analysis of The expression and clinical characteristics of ABCA1, A key gene in lipid droplet metabolism

**Figure 8:**
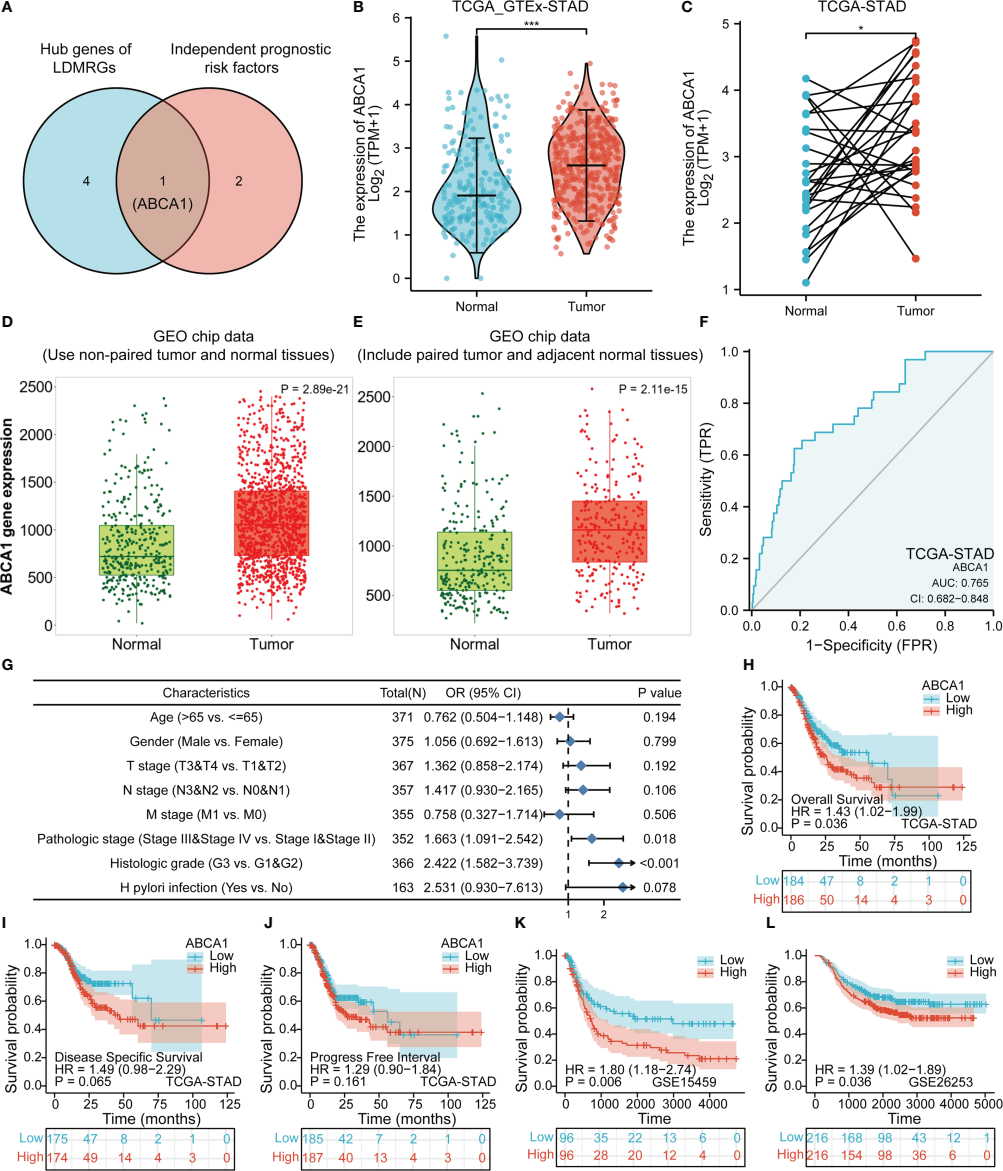
Expression and prognostic analysis of ABCA1 in gastric cancer. **(A)** Identification of the key gene, ABCA1, in LDMRGs in STAD. **(B–E)** Differential expression analysis of ABCA1 in gastric cancer using TCGA and GEO databases. **(F)** The diagnostic value of ABCA1 expression in gastric cancer was analyzed by ROC curve in STAD. **(G)** The correlation between ABCA1 expression and clinical characteristics was analyzed by logistic regression in STAD. **(H–J)** Correlation analysis of ABCA1 expression and survival prognosis in the TCGA-STAD cohort, including OS, DSS, and PFI. **(K, L)** Correlation analysis of ABCA1 expression and OS prognosis in the GSE15459 and GSE26253 cohorts. (*: p ≤0.05, ***: p ≤ 0.001).

The Venn diagram showed that we took the intersection of hub genes of LDMRGs and independent prognostic risk factors to identify a key gene associated with lipid droplet metabolism in STAD, ABCA1 (**Figure 8A**). By combining TCGA,GTEx and GEO data sets, we found that ABCA1 expression was significantly up-regulated in gastric cancer tissues compared with normal gastric tissues (**Figures 8B–E**). As shown in **Figure 8F**, the ROC curve showed that the expression level of ABCA1 had certain diagnostic value for STAD (AUC = 0.765; CI: 0.682 - 0.848). Furthermore, we found that ABCA1 expression was closely related to pathological stage and histologic grade by logistic regression analysis (**Figure 8G**). In addition, we explored that patients with gastric cancer in the ABCA1 high expression group had a worse prognosis in the TCGA-STAD cohort, the GSE15459 cohort and the GSE26253 cohort (**Figures 8H–L**). These results suggested that ABCA1 may promote gastric cancer progression.

### Functional enrichment analysis and correlation analysis of ABCA1 expression and immune infiltration and ICB treatment response in STAD

**Figure 9:**
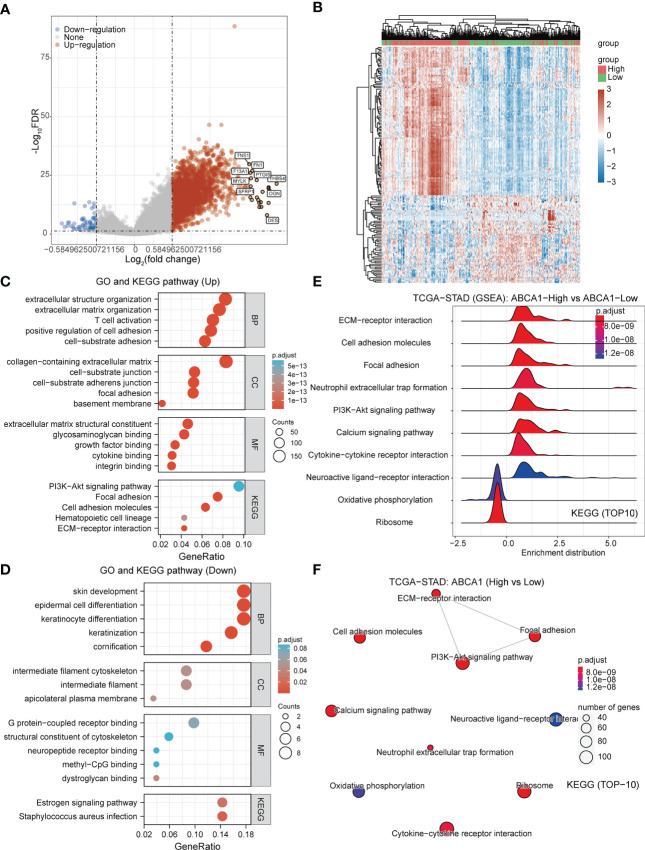
Gene function enrichment analysis of ABCA1 in STAD. **(A, B)** Volcano and heat maps of DEGs in the ABCA1 high and low expression groups in the TCGA-STAD cohort. **(C, D)** GO and KEGG pathway enrichment analysis of DEGs in STAD. **(E, F)** GSEA analysis based on ABCA1 expression in STAD.

To further explore the molecular functions played by ABCA1 in STAD, we divided the samples in the TCGA-STAD cohort into high and low expression groups based on the expression levels of ABCA1 and analyzed the DEGs between the two groups, including 2241 genes with up-regulated expression and 59 genes with down-regulated expression (**Figures 9A, B**). We then performed GO and KEGG pathway enrichment analysis as well as GSEA. The GO and KEGG pathway enrichment results showed that these differentially expressed up-regulated genes were mainly enriched in cell adhesion, T cell activation, and PI3K-Akt signaling pathways. Differentially expressed down-regulated genes were mainly enriched in cytoskeleton composition (**Figures 9C, D**). The GSEA results indicated that the KEGG pathway was mainly enriched in ECM-receptor interactions, cell adhesion, neutrophil extracellular trap formation, and PI3K-Akt signaling pathways (**Figures 9E, F**). These results suggested that ABCA1 may mediate cell adhesion through the PI3K-Akt signaling pathway and thus promote tumor metastasis, and further experiments are needed to verify the results.

**Figure 10:**
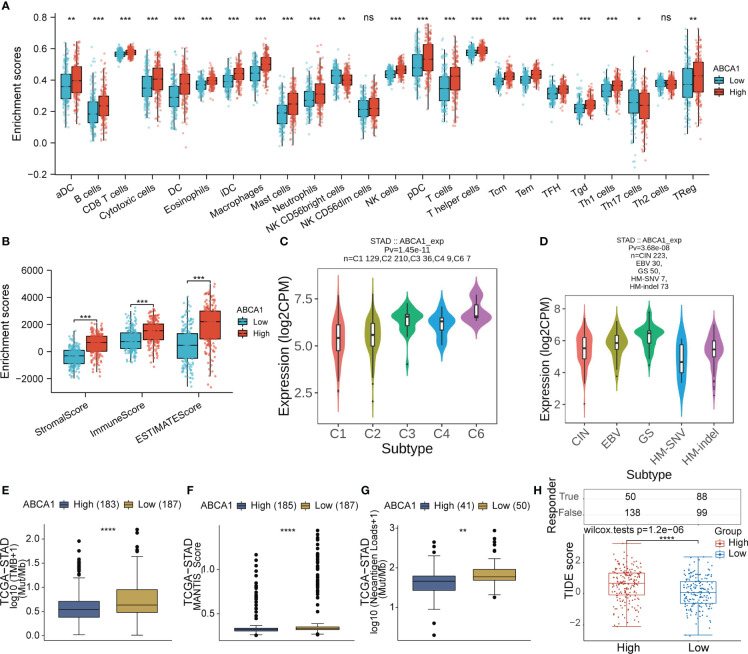
Correlation analysis of ABCA1 expression and immune characteristics in STAD. **(A, B)** Differential analysis of the level of immune infiltration between high and low expression groups of ABCA1 was performed in STAD by the ssGSEA and estimate algorithms. **(C, D)** Differential analysis of ABCA1 expression levels between different immunological and molecular subtypes in STAD using the TISIDB database. **(E-G)** Differential analysis of TMB, MSI, and neoantigen loads between high and low ABCA1 expression groups was performed in STAD using the CAMOIP database. **(H)** Differential analysis of TIDE scores between high and low ABCA1 expression groups in STAD. (ns: p > 0.05, *: p ≤0.05, **: p ≤ 0.01, ***: p ≤ 0.001, ****: p ≤ 0.0001).

In addition, immune cell infiltration analysis showed that high expression of ABCA1 correlated with high levels of infiltration of multiple immune cells (**Figures 10A, B**). As shown in **Supplementary Figure 1**, we also assessed the correlation between ABCA1 expression and immune infiltration by the CIBERSORT algorithm, which showed that ABCA1 expression was associated with a variety of stromal cells, including M2-type macrophages, myeloid dendritic cells, and mast cells. The expression of ABCA1 is closely related to a variety of immune and molecular subtypes in STAD (**Figures 10C, D**). These results indicated that the high expression of ABCA1 may contribute to the progression of gastric cancer by mediating the body’s immune regulation. To assess the relationship between ABCA1 expression and immunotherapy response, we explored the correlation between ABCA1 expression and TMB, MSI, and Neoantigen Loads. As shown in **Figures 10E–G**, the group with high ABCA1 expression had lower TMB, MSI, and Neoantigen Loads, suggesting that these patients may not respond well to ICB therapy. Also, our TIDE algorithm showed that the ABCA1 high expression group had higher TIDE scores, indicating that these patients were less effective on ICB treatment (**Figure 10H**).

### Mutation analysis of ABCA1 and correlation analysis of ABCA1 mRNA expression levels and drug sensitivity in STAD

**Figure 11:**
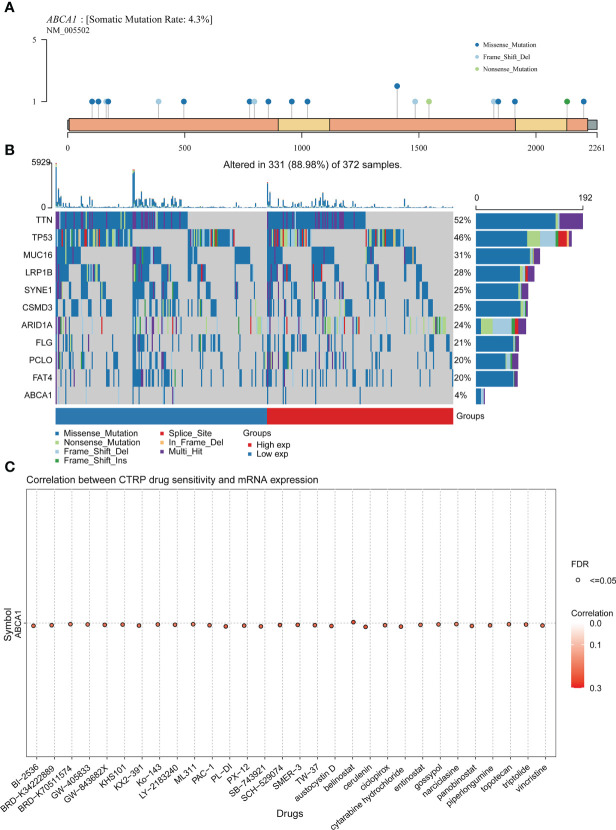
Mutation analysis and drug sensitivity analysis of ABCA1 in STAD. **(A)** Lollipop plot of ABCA1 mutation distribution in the genome. **(B)** A waterfall map of the somatic mutation landscape in the TCGA-STAD cohort, including the top 10 mutation-frequency genes and ABCA1. **(C)** Correlation analysis of ABCA1 mRNA expression and drug sensitivity in pan-cancer using the GSCA database.

Past research has shown that the accumulation of genetic mutations and tumour development are closely related (40). Our analysis showed a somatic mutation rate of 4.3% for ABCA1 and demonstrated the distribution of ABCA1 mutations in the genome in STAD. We noted that the somatic mutation type of ABCA1 was predominantly missense mutation (**Figure 11A**). As shown in **Figure 11B**, we analyzed the somatic landscape of the TCGA-STAD cohort and demonstrated the top 10 genes with the highest mutation frequency in the tumor samples by waterfall plots, including TTN, TP53, MUC16, LRP1B, SYNE1, CSMD3, ARID1A, FLG, PCLO, and FAT4 (**Figure 11B**).

In addition, we found a positive correlation between the mRNA expression levels of ABCA1 and the sensitivity of multiple drugs, suggesting that these chemotherapeutic agents may be more effective in patients with higher levels of ABCA1 expression (**Figure 11C**).

## Discussion

Gastric cancer has long been a worldwide public health problem with its high morbidity and mortality rates (2, 41). Especially for patients with advanced gastric cancer, chemotherapy-based monotherapy or combination therapy regimens have limited effect. In recent years, emerging targeted therapies have benefited only a small proportion of gastric cancer patients (42). Therefore, the identification of biomarkers that promote gastric cancer progression and the search for more effective therapeutic targets has been an ongoing clinical challenge.

Metabolic reprogramming, the ability of tumor cells to regulate energy metabolism to accelerate cell growth and proliferation, is also a characteristic of tumors (43). Tumor cells will choose the best mode for their own survival according to the microenvironment, and constantly change in the process of tumor development. Warburg effect is the starting point of the study of metabolic reprogramming in cancer. Current research has found that metabolic reprogramming is also involved in amino acid and lipid metabolism, with lipid metabolic reprogramming playing an important role in tumour progression (44). A marked increase in the *de novo* synthesis of fatty acids in tumour cells is accompanied by a marked enhancement of fatty acid oxidation to meet the demands of rapid tumour cell growth (45). In addition, many lipid signalling molecules, including phosphatidylinositol-3,4,5-trisphosphate, lysophospholipids, prostaglandins, and platelet-activating factors, which contribute to tumour progression by participating in signal transduction cascade reactions, in turn, contribute to tumour progression (46–48). Thus, the pathway regulating lipid metabolic reprogramming has emerged as an important potential target for tumour therapy (49).

Lipid droplets play an important role in the process of lipid metabolism and are the central hub for processing lipids (48). Lipid droplets are spherical monolayer organelles that primarily regulate lipid metabolism, transport and signalling in cells and tissues (50). Lipid droplet biogenesis is induced by nutritional and oxidative stress, and cancer cells promote tumour progression through the accumulation of lipid droplets to ensure energy production, redox homeostasis, and drive membrane synthesis (10). A study has constructed a risk prediction scoring model based on genes related to lipid metabolism, which can effectively predict the prognosis of patients (51). Lipid droplets are an important energy reservoir for cancer cells and accumulation of lipid droplets can be found in many cancer cells (52–55). Several studies have shown that autophagy and lipid droplet synthesis are closely related and that it can promote lipid droplet synthesis to increase the resistance of cancer cells to stress, thus promoting cancer cell progression (56). In gastric cancer, excess lipids are converted to triglycerides and cholesteryl esters in the ER, and the rate of fat synthesis increases, leading to the formation of lipid droplets. At the same time, the increased level of mitochondrial fatty acid β-oxidation not only provides sufficient energy for the growth and metastasis of cancer cells, but also participates in the transduction of lipid rafts and lipid modified signaling molecules, which further promotes tumor progression. In addition, drug resistance in gastric cancer may also be related to lipid metabolism (57, 58). However, no article has yet reported on the construction of risk models for genes related to lipid droplet metabolism in gastric cancer to predict survival prognosis and immunotherapy efficacy in patients with gastric cancer.

In this study, we first systematically analysed the multi-omics data of the LDMRGs. We found that most LDMRGs were differentially expressed in STAD and that there was generally a positive correlation between their expression. We constructed PPI interaction networks between LDMRGs and identified the top 5 hug genes and 2 important sub-networks. The gene function enrichment analysis revealed that LDMRGs are primarily involved in the metabolism of lipid droplets, including the storage, localization, and transport of lipids, lipoproteins, celiac particles, and cholesterol. In addition, we analyzed the genetic alteration landscape of LDMRGs and found that TMB,MSI was significantly higher in the genetically altered group of patients with STAD than in the genetically unaltered group. This meant that patients with STAD in the genetically altered group may be more sensitive to treatment with ICB. Furthermore, we found that the expression levels of most LDMRGs were positively correlated with CNV and negatively correlated with methylation levels in STAD. Notably, the expression levels of LDMRGs were strongly correlated with the level of multiple immune cell infiltration, which suggested that these genes were also involved in the regulation of immunity in STAD.

Furthermore, we classified the samples in the TCGA-STAD cohort into C1 and C2 subtypes based on the expression of LDMRGs and found that the C2 subtype had a worse survival prognosis. Gene functional enrichment analysis revealed that DEGs in the 2 subtypes were mainly enriched in immune regulation and cell adhesion. Differences in immune infiltration levels, TIDE scores, m6A methylation, and tumour stemness were also compared between the 2 subtypes. The results showed that the C2 subtype had higher levels of immune infiltration and expression of immune checkpoint-related genes, but the TIDE score showed that the C2 subtype had a higher score, suggesting that the C2 subtype was less effective in ICB treatment. We speculated that the C2 subtype may be more susceptible to tumour invasion and metastasis and insensitivity to ICB treatment due to dysregulation of cell adhesion and immune regulation, thus leading to a poorer survival prognosis.

To further explore the relationship between LDMRGs expression and prognosis of patients with STAD, we constructed lasso regression model based on the expression profiles of LDMRGs in STAD. Moreover, we validated the risk prediction scoring system with the GSE15459 and GSE66229 datasets and the results showed that the prediction model has reliable predictive efficacy for the OS prognosis of patients with gastric cancer. We noted that 8 genes screened by lasso regression analysis were associated with a variety of cancers in past studies, including hepatocellular carcinoma (59), prostate adenocarcinoma (60), chronic lymphocytic leukemia (61), pancreatic adenocarcinoma (62), and colon adenocarcinoma (63).To improve the clinical applicability of the model, we incorporated the prognostic molecules screened by the lasso regression analysis into the cox regression analysis and constructed a nomogram prediction model in STAD. We then evaluated the predictive efficacy of the model by a variety of methods and the results demonstrated that the model has a certain predictive efficacy for survival prognosis at 1, 3 and 5 years for patients with STAD.

Furthermore, we further identified a key prognostic molecule in LDMRGs in STAD, ABCA1. Past studies have shown that ABCA1 is a lipid transporter protein that plays an important role in maintaining HDL biosynthesis and cellular cholesterol homeostasis (64). Numerous studies have demonstrated that ABCA1 was associated with the development of a variety of cancers, including colon cancer (65, 66), myeloproliferative neoplasms (67), ovarian cancer (68, 69), prostate cancer (70), and melanoma (71). ABCA1 may have a dual role in cancer, with ABCA1 showing anti-cancer effects in breast and prostate cancers, but pro-cancer effects in colorectal, bladder and melanoma cancers (72). However, there are few reports of ABCA1 being associated with gastric cancer. Our results showed that the expression level of ABCA1 was closely related to the survival prognosis, pathological stage and histological grade of patients with STAD. The gene function enrichment results showed that ABCA1 is mainly involved in cell adhesion and PI3K-Akt signaling pathway. Past studies have shown that the PI3K-Akt signaling pathway was involved in the invasion and metastasis of a variety of cancers, including hepatocellular carcinoma (73), gastric cancer (74, 75), lung adenocarcinoma (76), colorectal cancer (77), and renal cell carcinoma (78). We speculated that ABCA1 may be involved in the invasion and metastasis of gastric cancer through the PI3K-Akt signaling pathway. Moreover, previous studies showed PI3K/Akt/mTOR signaling pathway as an important signaling pathway in lipid metabolism in gastric cancer (57, 79). In addition, our study revealed that ABCA1 expression in STAD was closely associated with immune infiltration, MSI, TMB, and neoantigen loads. Patients with STAD in the high ABCA1 expression group may be less effective in the treatment of ICB. Finally, mutational landscape analysis showed that the somatic mutation type of ABCA1 was mainly missense mutation and ABCA1 expression was associated with mutations in TP53. These results suggest that ABCA1 may promote gastric cancer progression through immune regulation and mutations in TP53. The expression level of ABCA1 was positively correlated with the sensitivity of most chemotherapeutic drugs, which meant that patients with high expression of ABCA1 may respond better to these chemotherapeutic drugs. Our study demonstrated that ABCA1 was closely related to the prognosis and immune regulation of patients with gastric cancer and could potentially be a new therapeutic target for gastric cancer.

However, there are some limitations to our study. Firstly, our analysis is mainly based on multiple online databases and lacks validation from a large external clinical multicentre cohort of gastric cancer. Secondly, our study was mainly conducted by bioinformatics analysis and lacks validation from basic cellular and animal experiments. Therefore, we will improve these deficiencies and further explore the mechanism of LDMRGs in gastric cancer in future studies.

## Conclusion

In summary, we identified 2 molecular subtypes based on the expression of LDMRGs and analyzed the survival prognosis, functional enrichment analysis, and immune status between the different molecular subtypes. In addition, we constructed lasso regression models and performed iterative validation on the GEO dataset with consistent results. A nomogram containing the prognostic molecules screened by the lasso regression analysis was generated, which improved the predictive value and clinical applicability of the model. Finally, we identified a key gene in LDMRGs, ABCA1, and analyzed the prognostic value of ABCA1 in gastric cancer by multi-omics. The results showed that ABCA1 was closely associated with multiple clinical features, immune infiltration, and drug sensitivity in gastric cancer patients. The present study provided evidence for the prognostic value of LDMRGs in gastric cancer and contributes to the development of diagnostic and prognostic biomarkers and therapeutic agents for patients with gastric cancer.

## Data availability statement

The datasets presented in this study can be found in online repositories. The names of the repository/repositories and accession number(s) can be found in the article/**Supplementary Material**.

## Author contributions

ML was primarily responsible for the design of the study protocol and writing of the manuscript. XF and HW contributed to the data organization and analysis. RJ and QG downloaded the data. ZC and QR contributed to the revision and review of the manuscript. YW and YZ contributed to the supervision of the study. All authors contributed to the article and approved the submitted version.

## Supplementary material

Supplementary Figure 1 The correlation of ABCA1 expression and immune cell infiltration assessed by CIBERSORT in STAD. **(A)** Heat map of the correlation between ABCA1 expression and immune infiltration. **(B)** The percentage abundance of each type of tumour-infiltrating cell in each sample in the STAD.

Supplementary Figure 2 OPLS-DA analysis based on the expression of LDMRGs in STAD. **(A)** Inertia bar plot for OPLS-DA model. **(B)** Permutation test plot for OPLS-DA model validation. **(C)** Score plot for the OPLS-DA model. **(D)** S-plot plot for OPLS-DA model.

Supplementary Table 1 Screening of lipid droplet metabolism-related genes using the GeneCards database.
