# Supplementary figures and images for: Characterization of lipid droplet metabolism patterns identified prognosis and tumor microenvironment infiltration in gastric cancer
Source: Front Oncol. 2023 Jan 11;12:1038932. doi: 10.3389/fonc.2022.1038932 (PMC9875057; doi:10.3389/fonc.2022.1038932)

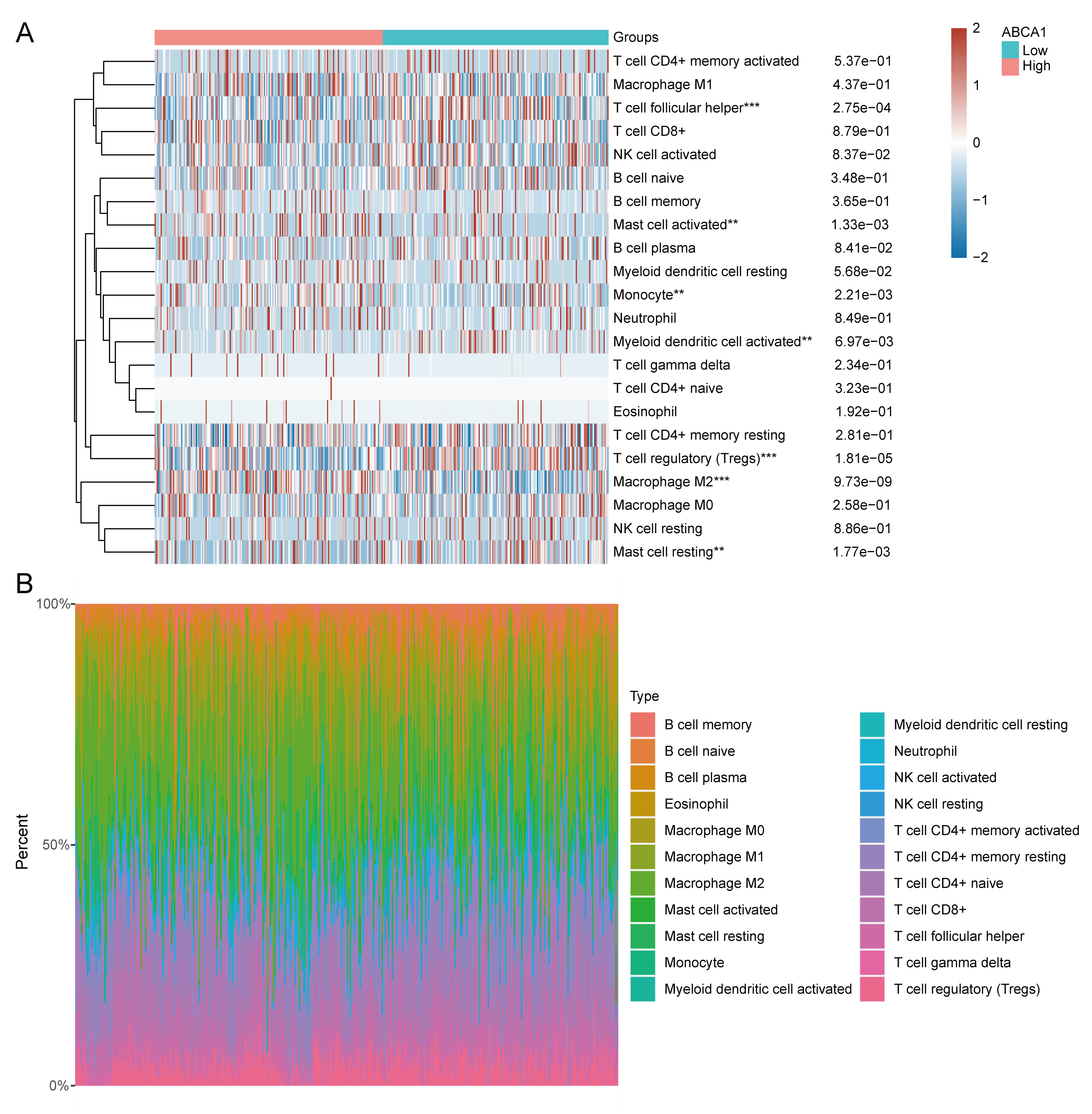

Supplement: Supplementary Figure 1 — The correlation of ABCA1 expression and immune cell infiltration assessed by CIBERSORT in STAD. (A) Heat map of the correlation between ABCA1 expression and immune infiltration. (B) The percentage abundance of each type of tumour-infiltrating cell in each sample in the STAD. [file Image_1.tif]

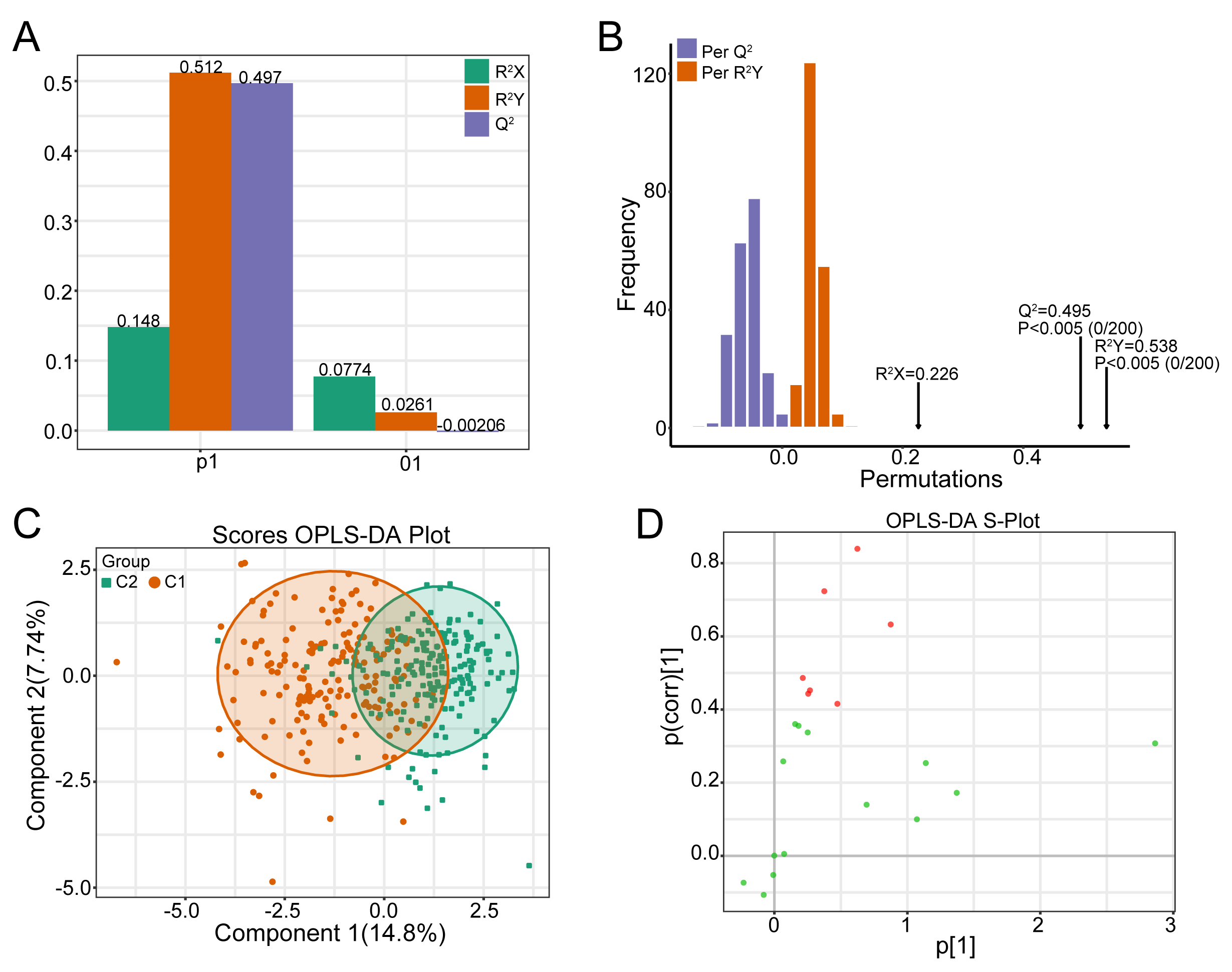

Supplement: Supplementary Figure 2 — OPLS-DA analysis based on the expression of LDMRGs in STAD. (A) Inertia bar plot for OPLS-DA model. (B) Permutation test plot for OPLS-DA model validation. (C) Score plot for the OPLS-DA model. (D) S-plot plot for OPLS-DA model. [file Image_2.tif]
