# Supplementary material for: Characterization of lipid droplet metabolism patterns identified prognosis and tumor microenvironment infiltration in gastric cancer
Source: Front Oncol. 2023 Jan 11;12:1038932. doi: 10.3389/fonc.2022.1038932 (PMC9875057; doi:10.3389/fonc.2022.1038932)
Supplement: Supplementary file 4 [file DataSheet_1.docx]

Links to the relevant raw data and R language code for this article (https://www.jianguoyun.com/p/DenUkUcQyOn0Chjd6OwEIAA).
